# Surface Layer Engineering of 3D-Printed PET-G: Effect of Printing Parameters on Surface Roughness and Wettability—Considerations for Tribological Applications

**DOI:** 10.3390/ma19173763

**Published:** 2026-09-04

**Authors:** Aleksandra Mirowska, Piotr Długosz, Michał Dobrzyński

**Affiliations:** Faculty of Mechanical Engineering and Ship Technology, Gdańsk University of Technology, Narutowicza 11/12, 80-233 Gdańsk, Poland; s185991@student.pg.edu.pl (P.D.); michal.dobrzynski@pg.edu.pl (M.D.)

**Keywords:** 3D printing, FDM, PET-G, surface topography, surface measurements, wettability

## Abstract

The aim of this study was to investigate the effect of 3D printing parameters on the surface topography and surface wettability properties of PET-G samples intended for potential tribological applications. Surfaces produced with varying fill rates and two layer heights were examined using focus variation microscopy and water contact angle measurements. The statistical analysis showed that the infill percentage has a significant influence on wettability, but did not reveal a significant influence of layer height on the observed contact angles. The results showed that surface characteristics are strongly dependent on process conditions, and the relationships between roughness parameters and wettability are mostly nonlinear. The parameters Są and Sq tended to decrease the contact angle as roughness increased, whereas Sp, Sv, and Sz showed no clear relationship with contact angle values. In contrast, Ssk and Sku showed a positive, linear correlation with the contact angle, indicating an important role of the statistical shape of the surface height distribution. The results confirm that the functional properties of additively manufactured surfaces depend not only on the amplitude of surface irregularities but also on their spatial organization and asymmetry.

## 1. Introduction

Additive manufacturing technologies, particularly fused deposition modeling/fused filament fabrication (FDM/FFF), are currently among the most rapidly developing methods for producing polymer components, as they combine relatively low process costs, great design freedom, and the ability to produce parts with complex geometries without the need for traditional tooling [1,2]. With the transition from prototyping to functional applications, increasing importance is being placed not only on dimensional accuracy and mechanical strength, but also on the quality of the surface layer, which—in the case of additively manufactured materials—is directly related to the characteristic topography of the deposition paths, porosity, structural anisotropy, and local process defects [3,4]. PET-G holds a special place among the filaments used in FDM technology; due to its favorable combination of good processability, high interlayer adhesion, chemical resistance, and satisfactory mechanical stability, it is used in the production of functional parts, cases, guide rails, technical components, and parts exposed to contact with working media [5,6,7]. At the same time, the literature emphasizes that the surface properties of PET-G prints are highly dependent on process parameters such as layer height, path orientation, print speed, extrusion temperature, and infill density, as these factors determine the geometry of the deposited material strands, their mutual interaction, and the final surface finish [8,9].

These issues are particularly significant in tribological applications, where the condition of the surface layer determines the actual contact area, pressure distribution, friction initiation mechanism, lubricant retention capacity, and wear resistance [10]. In the case of 3D-printed polymers, numerous studies indicate that the layered structure and the anisotropy of the printing process can lead to significant changes in the coefficient of friction and wear rate, as well as to a directional dependence of performance on the print trajectory [11,12]. In this context, PET-G is a particularly interesting material because, while it shows potential for use in lightweight sliding systems and low-maintenance motion mechanisms, its tribological properties remain less well understood than those of engineering polymers such as PA or PEEK. Previous studies have shown that the tribological properties of PET-G can be improved by modifying its composition, including through the addition of graphene or integration with PTFE, which leads to a reduction in the coefficient of friction and improved wear resistance [13,14]. At the same time, increasing attention is being paid to the wettability of additively manufactured surfaces, as the contact angle can be considered an indirect indicator of a surface’s ability to interact with liquids, the flow of the lubricant, and the creation of conditions conducive to boundary or mixed lubrication [15,16]. Theoretically, the interpretation of these phenomena is based primarily on the Wenzel and Cassie–Baxter models, which describe the influence of surface roughness and topographic heterogeneity on the apparent contact angle, indicating that roughness can enhance both the hydrophilic and hydrophobic properties of a surface, depending on the state of contact between the droplet and the surface [17].

Despite the rapid advancement of research in 3D printing, there are still gaps in the state-of-the-art. A significant part of the literature focuses on mechanical strength, dimensional accuracy, or general roughness values, while relatively fewer studies have addressed the relationships between specific surface topography parameters and the wetting properties and tribological potential of PET-G surfaces. This applies in particular to parameters such as Sa, Sq, Sp, Sv, Sz, Ssk, and Sku, which describe not only the amplitude of surface irregularities but also the statistical nature of the height distribution and the presence of topographic extremes. In the context of wettability, this is of crucial importance, because the static contact angle depends not only on how rough the surface is, but primarily on the mechanism of interaction between the liquid and the topography. A negative Ssk can favor air trapping and the transition to the Cassie–Baxter regime [18], while positive Ssk increases the number of edges and asperities, which affects contact line pinning. High Sku indicates more sharp features that stabilize the meniscus and increase the hysteresis of the contact angle. Only by understanding the impact of a comprehensive set of parameters describing surface texture will it be possible to determine its effect on the surface’s functional properties, including its wettability [19,20]. Furthermore, the relationship between these correlations and the sample printing parameters, as well as the significance of the infill percentage and layer height in shaping the surface layer of PET-G in the context of future applications involving sliding and components that interact with working fluids, also remains insufficiently studied.

For this reason, the aim of this study is to determine the influence of selected 3D printing parameters such as layer height and infill percentage on the surface topography of PET-G and its wettability. These results have been further discussed in the context of potential tribological applications. One of the objectives of this study was to investigate surface topography and wettability as potential descriptors of prospective tribological performance. This topic is both timely and significant from a scientific perspective as it contributes to our understanding of the relationship between surface microgeometry and fluid behavior on additive-manufactured parts and from an applied perspective, as it can support the design of the surface layer of components intended for operation in conditions involving friction, wear, and interaction with working fluids.

## 2. Materials and Methods

### 2.1. Material and Modelling

**Figure 1:**
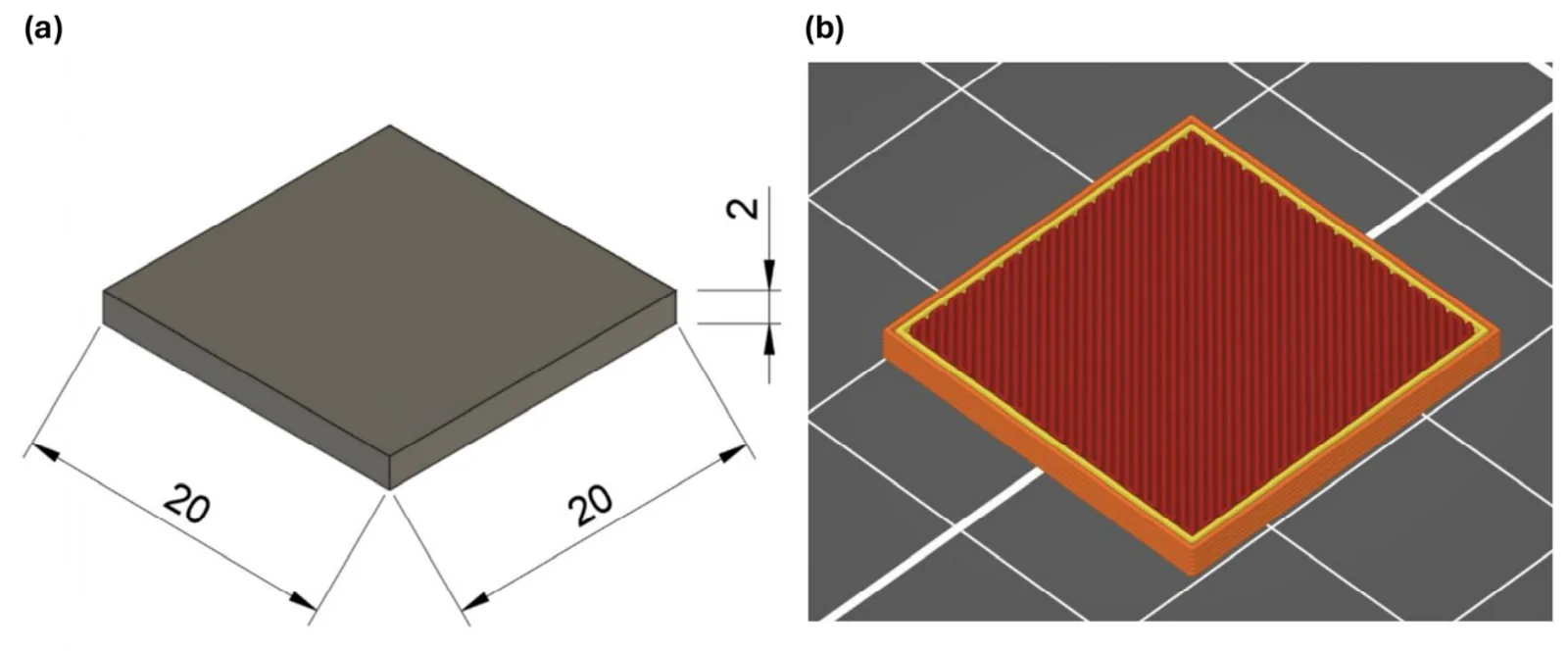
A geometric model of the samples prepared for printing (**a**) and a model divided into layers, showing the nozzle paths during the printing of subsequent layers (**b**).

The research employed PET-G (polyethylene terephthalate glycol) to conduct these studies. This material is defined as thermoplastic copolyester made from terephthalic acid, ethylene glycol, and a second glycol modifier called 1,4-cyclohexanedimethanol (CHDM). A 1.75 mm diameter PET-G filament (Fiberlogy, Brzezie, Poland) was used to prepare the samples. The sample models were designed in a CAD environment using Autodesk’s Fusion 360 software. A simple sample geometry was proposed, and the dimensions were selected to ensure an adequate measurement surface while minimizing the risk of manufacturing defects, such as the sample detaching from the printer bed during the cooling process. The model and geometry of the specimens are shown in Figure 1a.

### 2.2. Printing

The samples were produced utilizing the fused deposition modeling (FDM) method with a Prusa Core One 3D printer (Prusa Research, Prague, Czech Republic). To prepare the sample model, it was subjected to a slicing process in PrusaSlicer 2.9.6 (Prusa Research, Prague, Czech Republic) during which the manufacturing parameters were defined. Figure 1b shows an example model divided into layers and a visualization of the nozzle paths during the printing of subsequent layers. The following printing parameters were specified: nozzle diameter 0.4 mm, printing speed 80 mm/s, layer width 0.4 mm, and nozzle temperature 225 °C. The samples were produced using various layer heights (0.30 and 0.35 mm) and infill percentages (from 80% to 100%). The ranges of these printing parameters were selected based on preliminary studies and the recommended values presented in the literature [21,22,23]. All of the samples were prepared with a rectilinear infill pattern, bed temperature of 80 °C, raster orientation 0° in relation to the surfaces under investigation, with seam position aligned and with no active part cooling. The samples were produced with identical parameters characterized with two wall lines, two top and two bottom layers. The extrusion multiplier was set to 1.00. The PET-G filament was stored in its original factory-sealed, moisture-barrier packaging with desiccant and was opened immediately before printing; no additional drying step was applied, and no signs of moisture-related extrusion instability or surface defects were observed during specimen fabrication. Table 1 provides the sample designations with the defined values for these parameters. For each combination of the parameters N = 3 were investigated in the studies. All of the samples were printed from the same spool and under the same environmental conditions.

### 2.3. Macro- and Microscopic Observations

After the printing process was completed, the samples were removed from the working platform and subjected to an initial visual assessment. In order to preserve the actual surface structure resulting from the manufacturing process, the samples were not subjected to any additional finishing treatments, such as grinding or polishing. The analysis focused on visible material paths, the uniformity of the deposited layers, the presence of a stair-step effect, and any discontinuities, excess material, or surface defects resulting from the manufacturing process. Observations using an optical microscope (BX51, OLYMPUS, Tokio, Japan) allowed for an assessment of surface uniformity and the directionality of the structure resulting from the printhead’s movement trajectory.

### 2.4. Surface Topography Measurements

The geometric structure of the sample surfaces was analyzed using a non-contact method based on focus variation method on a Sensofar S neox 090 (Sensofar, Terassa, Spain) optical profilometer with a SensoMAP Standard 9 software. The measured area was 3.38 mm × 2.83 mm, with a pixel size of 2.76 μm; a Nikon EPI 5X objective lens with 5× magnification was used for this purpose. Measurements were taken in three selected areas for each sample, which allowed for the consideration of any surface inhomogeneity resulting from the 3D printing process. The measurement areas were selected to ensure representativeness, taking into account the direction of layer deposition and characteristic topographical features, such as material extrusion lines. Each measurement involved registering a surface within a specific field of view, followed by its analysis using filtering and data processing. Processing of the obtained image included:

- Form removal and leveling (LS-plane),
- Spectral filtering (a Robust Gaussian filter conforming to ISO 16610-31 [24]),
- Filling of non-measured points,
- Data visualization (three-dimensional surface topographic maps),
- Determination of roughness parameters in accordance with ISO 25178 [25] to quantify three-dimensional surface topography

### 2.5. Wettability Tests

The specimens were not subjected to any intentional storage or conditioning period, and contact angle measurements were performed immediately after printing to minimize potential changes in surface wettability resulting from environmental exposure. Surface wettability was characterized by static water contact angle measurements using the sessile drop technique. Approximately 3 µL droplets of deionized water were deposited onto the sample surface and analyzed with an optical tensiometer (Attention Theta Life, Biolin Scientific, Espoo, Finland). Contact angle values were determined using OneAttension software (Biolin Scientific, Finland), which calculates the droplet profile according to the Laplace–Young fitting model. For all measurements, the specimens were positioned consistently so that the water droplet was observed along the direction of the printed raster paths. Measurements were performed at ambient laboratory conditions (approximately 20 °C), with each recording lasting 10 s. The reported values represent the arithmetic mean of three measurements performed within different locations on each sample. A two-way analysis of variance (ANOVA) was performed, with statistical significance determined at *p* < 0.05.

## 3. Results and Discussion

**Figure 2:**
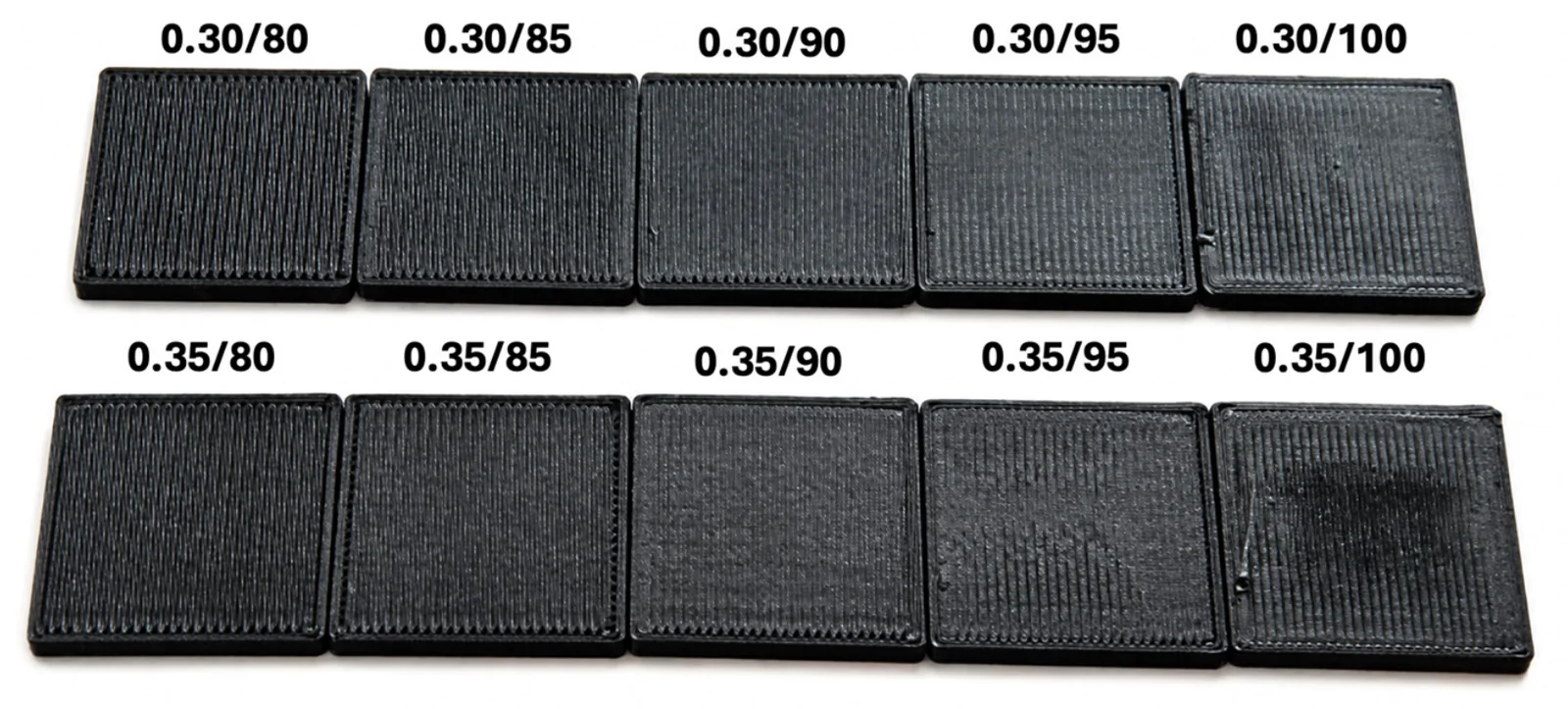
Macroscopic picture of the produced samples.

Figure 2 presents a macroscopic picture of the produced samples. Macroscopic analysis reveals no visual differences between samples with varying degrees of filling. The surfaces of all samples exhibit a uniform texture, with no visible defects such as delamination, bubbles, voids, or localized deformations. The absence of obvious discontinuities suggests proper interlayer adhesion and appropriately selected process conditions, including extrusion temperature and print speed.

Regardless of the fill rate or layer height used, the surface of the samples clearly exhibits the directionality characteristic of FDM technology, resulting from the print head’s movement trajectory. The material deposition lines are parallel and regular, which further confirms the stability of the process and the precise control of print parameters. The differences between samples produced at layer heights of 0.30 mm and 0.35 mm are subtle and are limited mainly to slight variations in the visibility of the material paths; however, they do not affect the overall macroscopic surface quality.

**Figure 3:**
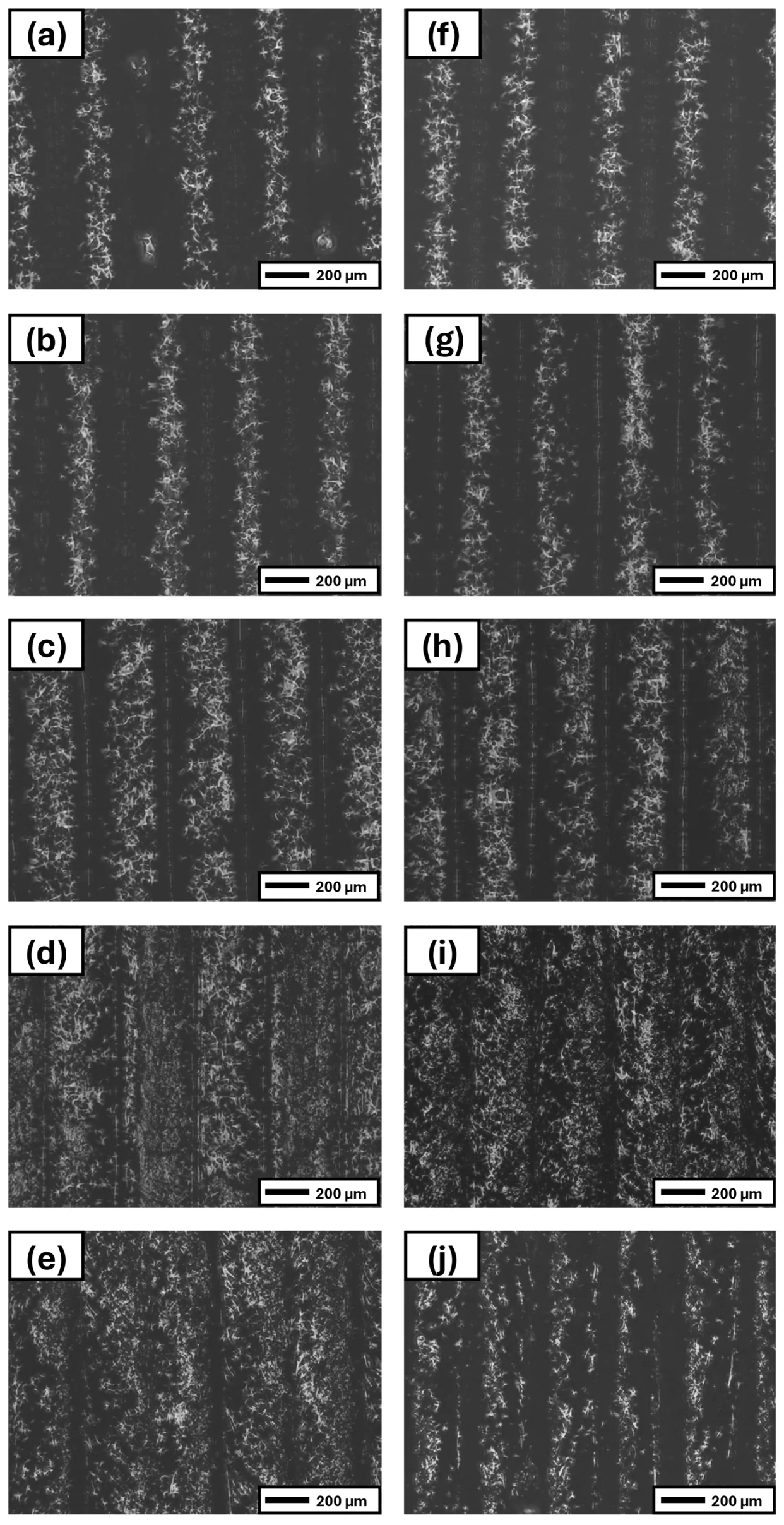
Optical microscope images of the samples produced with 0.30 mm (**a**–**e**) and 0.35 mm (**f**–**j**) layer height with 80 (**a**,**f**), 85 (**b**,**g**), 90 (**c**,**h**), 95 (**d**,**i**) and 100% (**e**,**j**) infill percentages.

The optical microscope images shown in Figure 3 reveal distinct differences in the internal structure of the samples, resulting from the varying percentages of infill used in the FDM printing process. In contrast to macroscopic observations, microscopic analysis allows for an unambiguous distinction between the geometry and density of the material layers within individual samples. As the infill density increases, a gradual reduction in the proportion of voids between adjacent material layers is observed, leading to a more compact and homogeneous structure. For samples with lower infill percentages (80–85%), distinct, regularly spaced pores and larger gaps between filaments are visible, resulting from the chosen packing strategy. At higher infill ratios (90–100%), the structure becomes denser, and the boundaries between individual material paths become less distinct, indicating better integration and greater material continuity.

**Figure 4:**
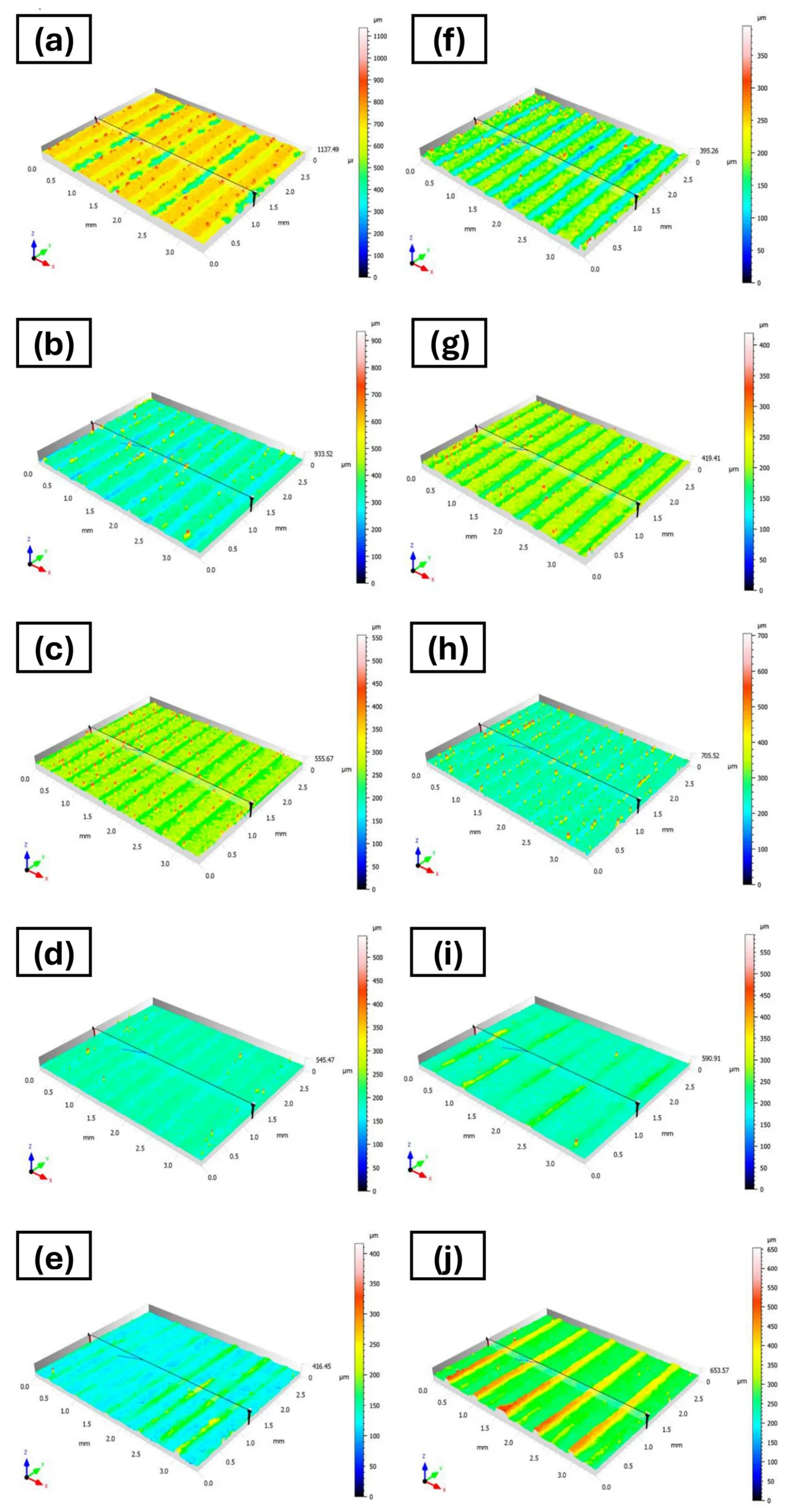
3D topographic maps of the samples produced with 0.30 mm (**a**–**e**) and 0.35 mm (**f**–**j**) layer height with 80 (**a**,**f**), 85 (**b**,**g**), 90 (**c**,**h**), 95 (**d**,**i**) and 100% (**e**,**j**) infill percentages.

The three-dimensional surface topography of the samples, as observed by focus variation microscopy, is shown in Figure 4. Images obtained for the analyzed PET-G samples produced using 3D printing show a clearly ordered surface morphology that is strongly correlated with the specified process parameters. Regardless of the combination of process parameters, the observed topography confirms the presence of characteristic material deposition paths that form a repeatable, anisotropic geometric pattern. This structure is consistent with observations made using optical microscopy; however, the use of focal variation microscopy allows for a more detailed analysis of height variations and local differences in roughness. As the infill percentage increases, a gradual increase in the density of the material paths and a reduction in the space between them become apparent. Surfaces with higher fill levels are characterized by a more uniform profile, with fewer local dips and limited presence of micropores. At the same time, no distinct defects such as delamination or discontinuities in the paths are observed, which indicates the stability of the manufacturing process and good interlayer adhesion. In the case of samples with lower infill levels, the structure remains more open, and height contrasts are more pronounced, resulting in greater heterogeneity of the surface topography. However, for samples with 100% filling, a different effect is observed in the form of increased surface area and more pronounced roughness. This phenomenon can be attributed to excessive compaction of the material tracks, which, given the limited ability of the plasticized polymer to relax, leads to their partial overlap and local accumulation of material. As a result, distinct ridges and irregularities form due to the accumulation of excess volume at the interfaces between adjacent strands. This mechanism may be further exacerbated by limited heat dissipation and reduced space for the free flow of material, which promotes the fixation of irregularities during solidification. The higher surface roughness observed at 100% infill may be associated with increased track overlap, local material accumulation, and greater compaction of the deposited material; however, these mechanisms remain plausible interpretations of the observed surface characteristics and were not directly verified in the present study. From a tribological perspective, this surface morphology can influence important functional implications. The increased roughness in samples with the highest degree of filling may potentially lead to intensified mechanical interactions at the interface between mating surfaces, increasing the contribution of adhesive and deformational friction mechanisms. At the same time, the presence of distinct, directionally aligned ridges may result in anisotropy of tribological properties, depending on the direction of motion relative to the print trajectory. In contrast, surfaces with moderate infill, characterized by a better balance between material compaction and relaxation capacity, may theoretically offer a more favorable compromise between contact load-bearing capacity and lubricant retention capacity.

**Figure 5:**
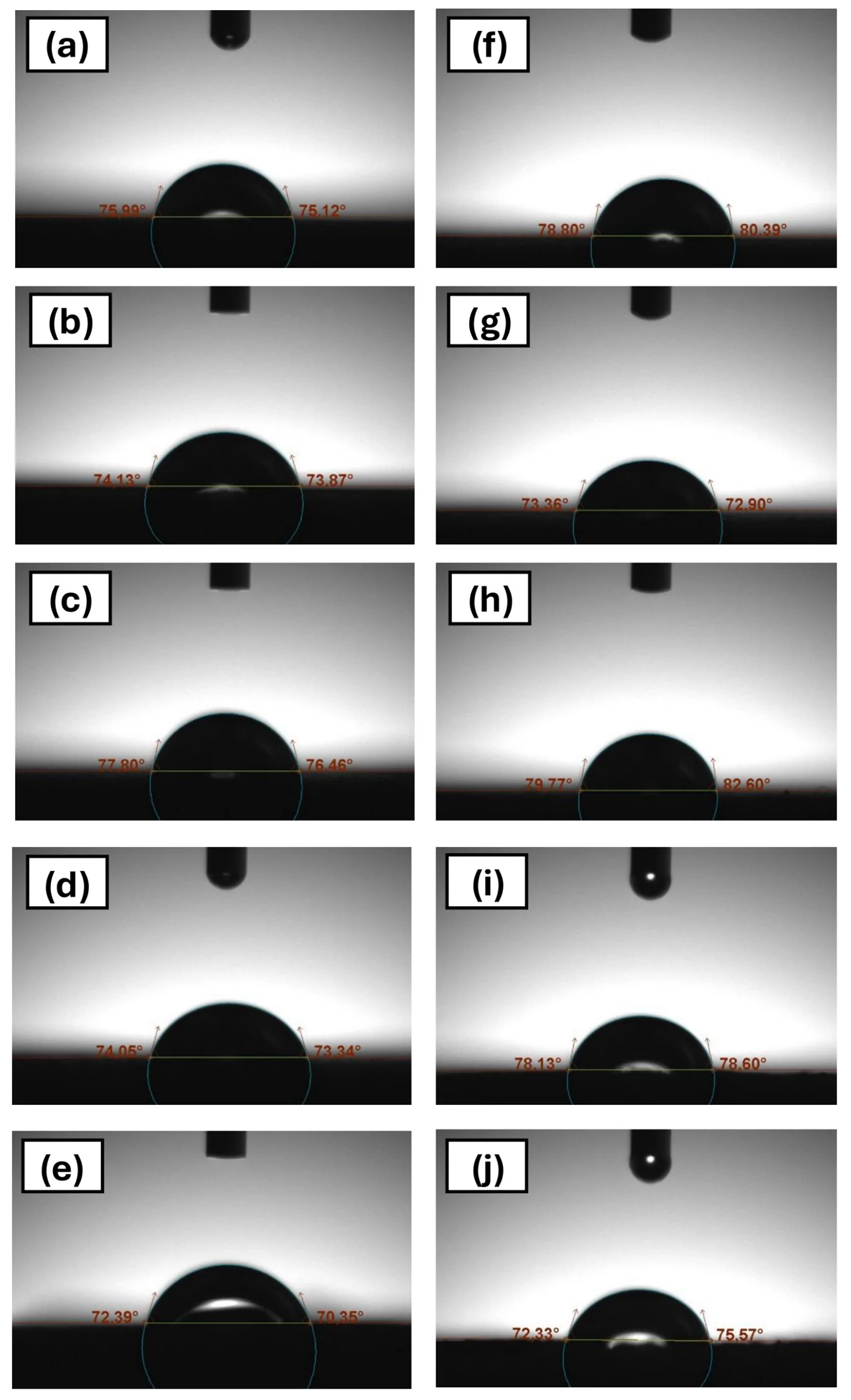
Measured contact angle values for all investigated samples produced with 0.30 mm (**a**–**e**) and 0.35 mm (**f**–**j**) layer height with 80 (**a**,**f**), 85 (**b**,**g**), 90 (**c**,**h**), 95 (**d**,**i**) and 100% (**e**,**j**) infill percentages.

The contact angle measurement provides insight into the surface wettability, which can be closely related to tribological performance of the surfaces due to the process of retaining lubrication. Table 2 presents contact angle values obtained for all of the samples and Figure 5 presents the images depicts during testing. Measurements of the water contact angle for the analyzed PET-G surfaces indicate that all tested samples are moderately hydrophilic, while showing relatively low variation among the different process variants. The obtained values fall within a narrow range, suggesting that changes in printing parameters affect wettability in a subtle and indirect manner, primarily by modifying the surface topography rather than its chemical nature. For the samples produced with a lower layer height, relatively stable contact angle values are observed, with a slight tendency for the angle to increase as the fill ratio is raised to intermediate levels. This may result from a more ordered and homogeneous surface structure, which helps to reduce the effective contact area between the liquid and the substrate. At the same time, a decrease in the contact angle is observed at maximum infill density, which is consistent with earlier topographical observations indicating an increase in surface area and local material agglomerations. Such irregularities may promote a transition toward a state more closely resembling the Wenzel model, in which the liquid penetrates the microstructure more effectively, thereby increasing effective wettability. A similar trend is observed for 0.35 mm thickness layers, where an increase in the contact angle is also observed for intermediate degrees of compaction, followed by a decrease for fully compacted samples. In this case, this effect may be further amplified by more pronounced macro- and micro-roughness resulting from the greater amplitude of the deposition tracks. Such a structure promotes an increase in capillary phenomena and the local anchoring of liquid droplets in surface irregularities. From the perspective of tribological applications, the observed differences in wettability may be important factor for the formation and stability of the lubricating film. Surfaces with slightly higher contact angles, associated with a more ordered topography, may exhibit limited liquid retention capacity, which favors boundary friction conditions. Conversely, surfaces with greater geometric complexity and lower contact angles can potentially more effectively retain the lubricant in micro-depressions, facilitating the possible transition to a mixed-friction regime. Consequently, as with roughness, the optimization of printing parameters should take into account the trade-off between geometric structure and surface properties that determine interaction with working fluids.

A two-way ANOVA was performed to determine statistical significance. The results of the analysis are presented in Table 3. The analysis considered five levels of infill ratio (80, 85, 90, 95, and 100%) and two levels of layer thickness (0.30 and 0.35 mm), with three independent replicates for each combination of parameters. The analysis revealed a statistically significant effect of the infill percentage on the measured contact angle (*p* < 0.05). However, no significant effect of layer height was found (*p* = 0.6154), nor was there a significant interaction between the filling percentage and layer height (*p* = 0.7815), indicating that the effect of the degree of filling on wettability did not depend significantly on the layer height proposed. The average values of the contact angle for filling levels of 80, 85, 90, 95, and 100% are presented in Table 2. Due to the significant main effect of the percentage of infill, a Tukey’s post-hoc test was conducted. The results of the analysis are presented in Table 4. Significant differences were found between the 85% and 90% levels (*p* = 0.0499), the 90% and 100% levels (*p* = 0.0203), and the 95% and 100% levels (*p* = 0.0239). The remaining comparisons did not reveal any statistically significant differences.

**Figure 6:**
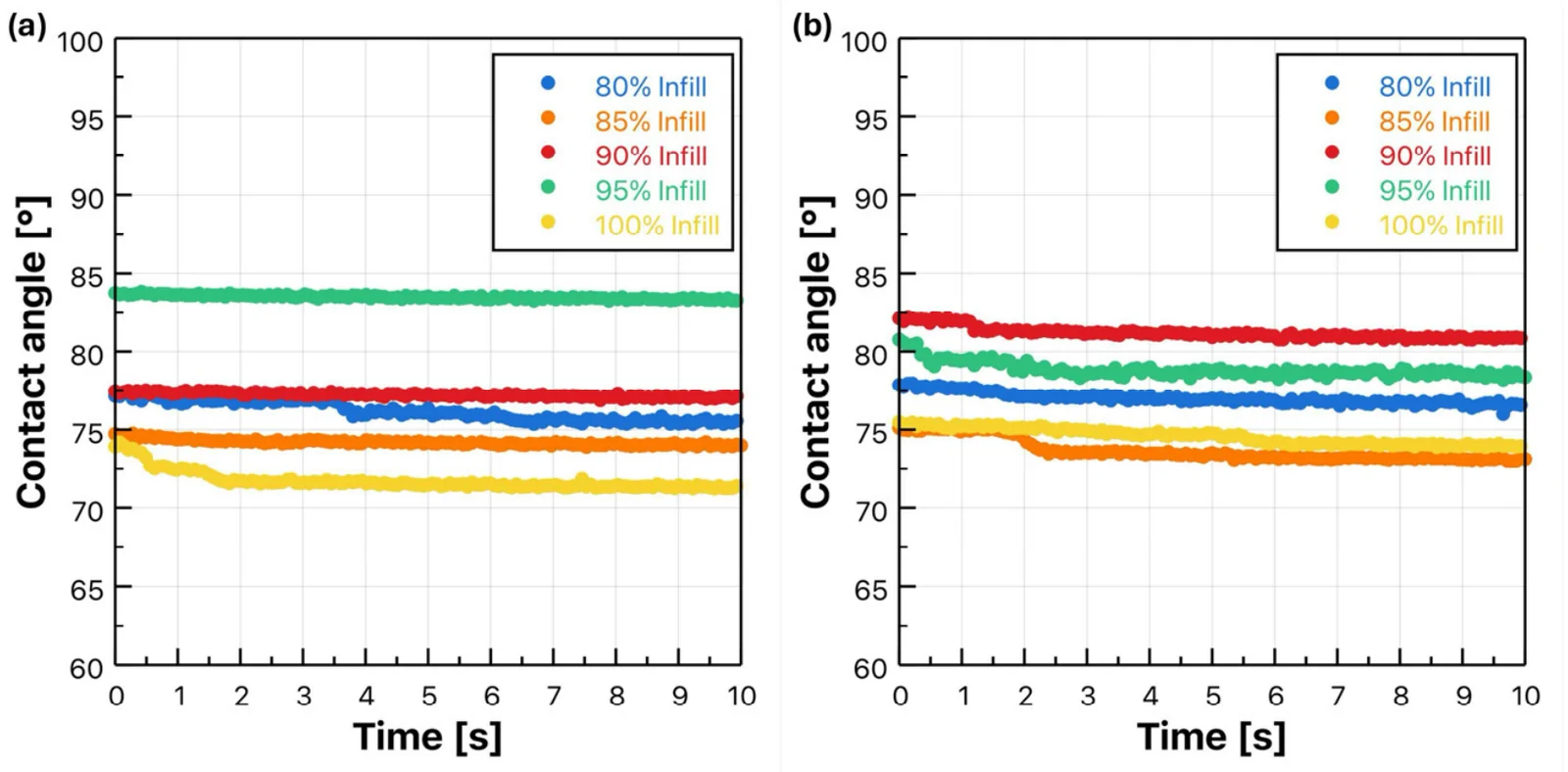
Contact angle values as a function of time for the samples produces with 0.30 mm (**a**) and 0.35 mm layer height (**b**).

The plots of the contact angle as a function of time shown in Figure 6 indicate that water droplets stabilize very quickly on the surface of the tested samples. Already in the initial phase of the measurement, the contact angle values reach an equilibrium state, and the subsequent trend is nearly constant, as reflected in the quasi-linear, horizontal course of the curves. This behavior suggests limited dynamics in the processes of liquid spreading and penetration into the surface structure, which can be attributed to the material’s relatively low absorbency and stable surface energy. Altay et al. [26] also observed almost immediate stabilization of the contact angle over time on a PET substrate for the water sessile drop method. From a tribological perspective, this means that wetting conditions stabilize practically immediately upon contact, which can theoretically promote the predictable formation of a liquid film during the initial phase of surface interaction.

**Figure 7:**
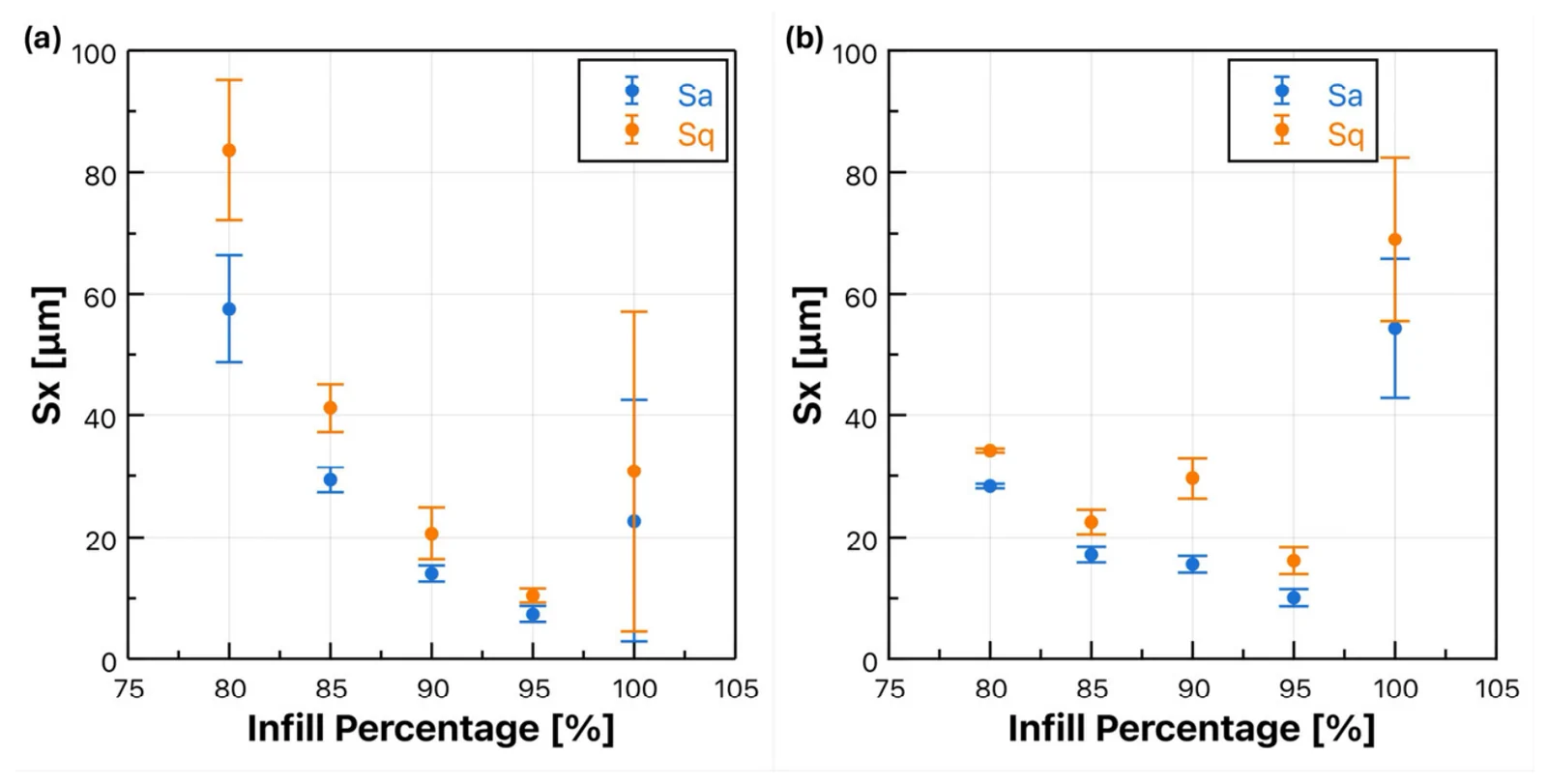
Sa and Sq parameters values as functions of infill percentages for the samples produced with 0.30 mm (**a**) and 0.35 mm layer height (**b**).

Figure 7 shows the relationship between Sa and Sq parameters and the percentage of infilling for layer heights of 0.30 mm (a) and 0.35 mm (b). Both graphs show a clear influence of the percentage of infilling on the Sa and Sq roughness parameters, with the relationship exhibiting a nonlinear trend. In both cases, the parameter values decrease as the structure’s density increases up to an intermediate level and reach their lowest values at 95% infill within the investigated range of printing parameters and specimen configuration, after which they rise again at 100% infill density. This is particularly evident for a layer height of 0.35 mm. The initial relationship is consistent with reports that increased packing density can improve the continuity of the resulting structure while simultaneously affecting the local surface topography [27]. At full infill, however, both parameters increase again, which can be attributed to excessive path density, their local overlap, and material accumulation at the contact zones, leading to greater topographic irregularity. Similar relationships for additively printed surfaces are described as the result of interactions between the geometry of the tracks, the flow of the plasticized polymer, and the solidification conditions [28,29]. It is worth noting that for both layer heights, the standard deviations for full infill are the largest, suggesting less stable surface formation under these conditions. From a tribological perspective, this relationship can be significant because the Sa and Sq parameters reflect the severity of surface irregularities that can influence friction and wear mechanisms. Surfaces with lower roughness in the intermediate filling range may promote more stable contact and fewer local stress concentrations, whereas surfaces with high values of these parameters, especially at full filling, can theoretically generate a higher proportion of adhesive friction. However, these relationships should be directly investigated by measuring the coefficient of friction and wear.

**Figure 8:**
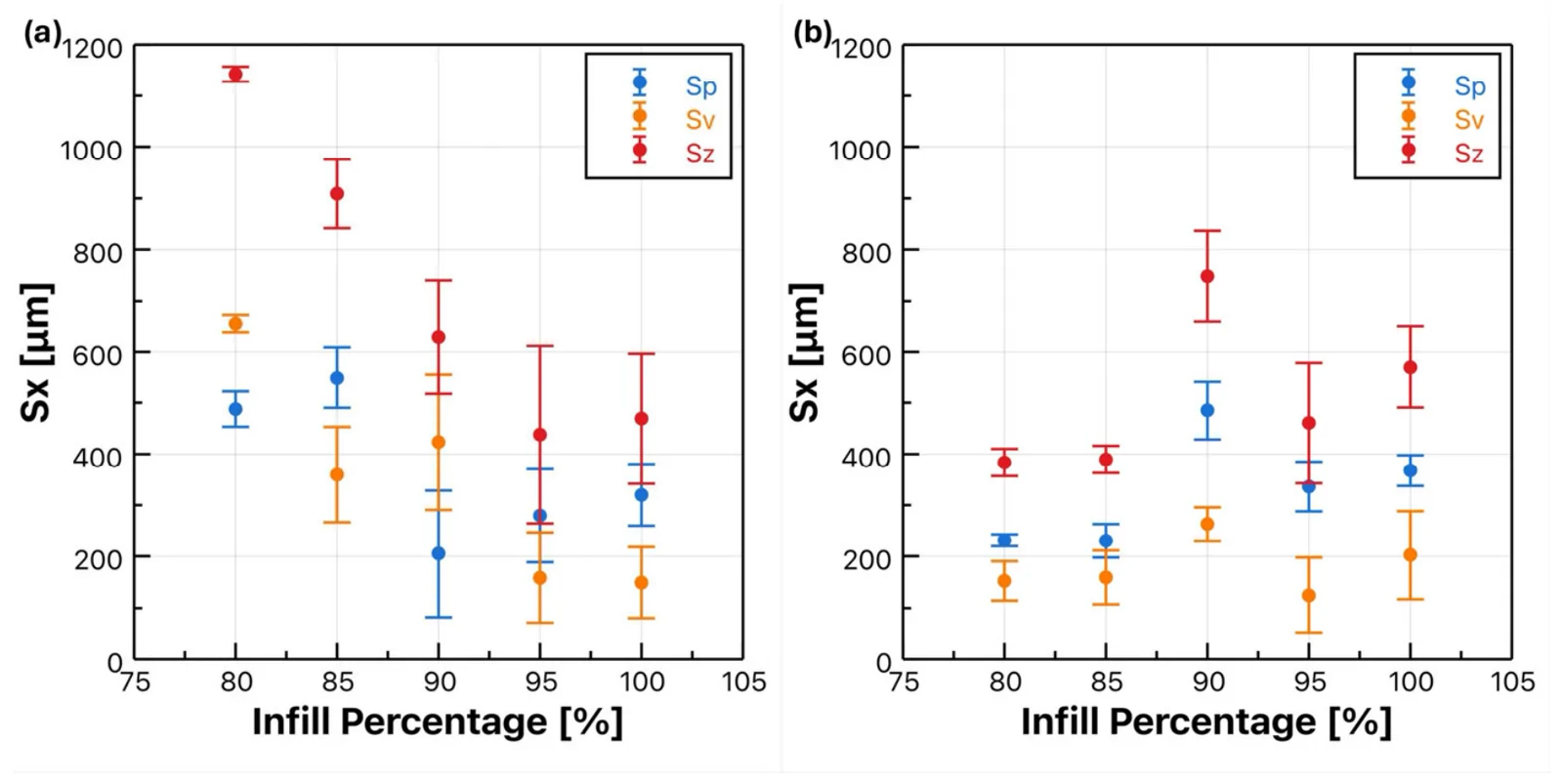
Sp, Sv and Sz parameters values as functions of infill percentages for the samples produced with 0.30 mm (**a**) and 0.35 mm layer height (**b**).

The relationship between the key roughness parameters—Sz, Sp, and Sv—in terms of infill density is shown in Figure 8. In the case of the Sz parameter, which describes the total topographic height, the observed relationships indicate a distinctly different effect of the filling degree depending on the layer thickness. For the 0.30 mm layer, the Sz value systematically decreased as the filling degree increased, suggesting a gradual reduction in the amplitude of irregularities and a more uniform surface profile, whereas for the 0.35 mm layer, the highest Sz values occurred at 90–100% infill, which can be interpreted as a result of more intensive layer deposition, greater profile amplitude, and a stronger influence of local material accumulations; this behavior is consistent with reports in the literature, according to which layer height and path geometry in FDM printing significantly determine the development of surface topography [30]. In contrast, for the Sp and Sv parameters, no clear dependence on the infill density was observed, suggesting that these changes did not predominantly affect either the highest peaks or the deepest valleys of the surface; this means that modifying the infill primarily affected the overall organization and density of surface irregularities, without, however, leading to a clear restructuring of topographic extremes, which is consistent with observations that not all height parameters respond equally to changes in additive manufacturing conditions. From a tribological perspective, this can provide important influence because the Sz parameter reflects the intensity of surface roughness, while Sp and Sv refer to local peaks and valleys that can potentially influence, respectively, contact initiation and lubricant retention. Consequently, a decrease in Sz at a 0.30 mm film thickness may promote more stable contact and lower stress concentrations, whereas higher Sz values at a 0.35 mm film thickness and greater fill ratio may increase the contribution of asperity interactions to friction, without a simultaneous increase in the extreme topographic regions described by Sp and Sv.

**Figure 9:**
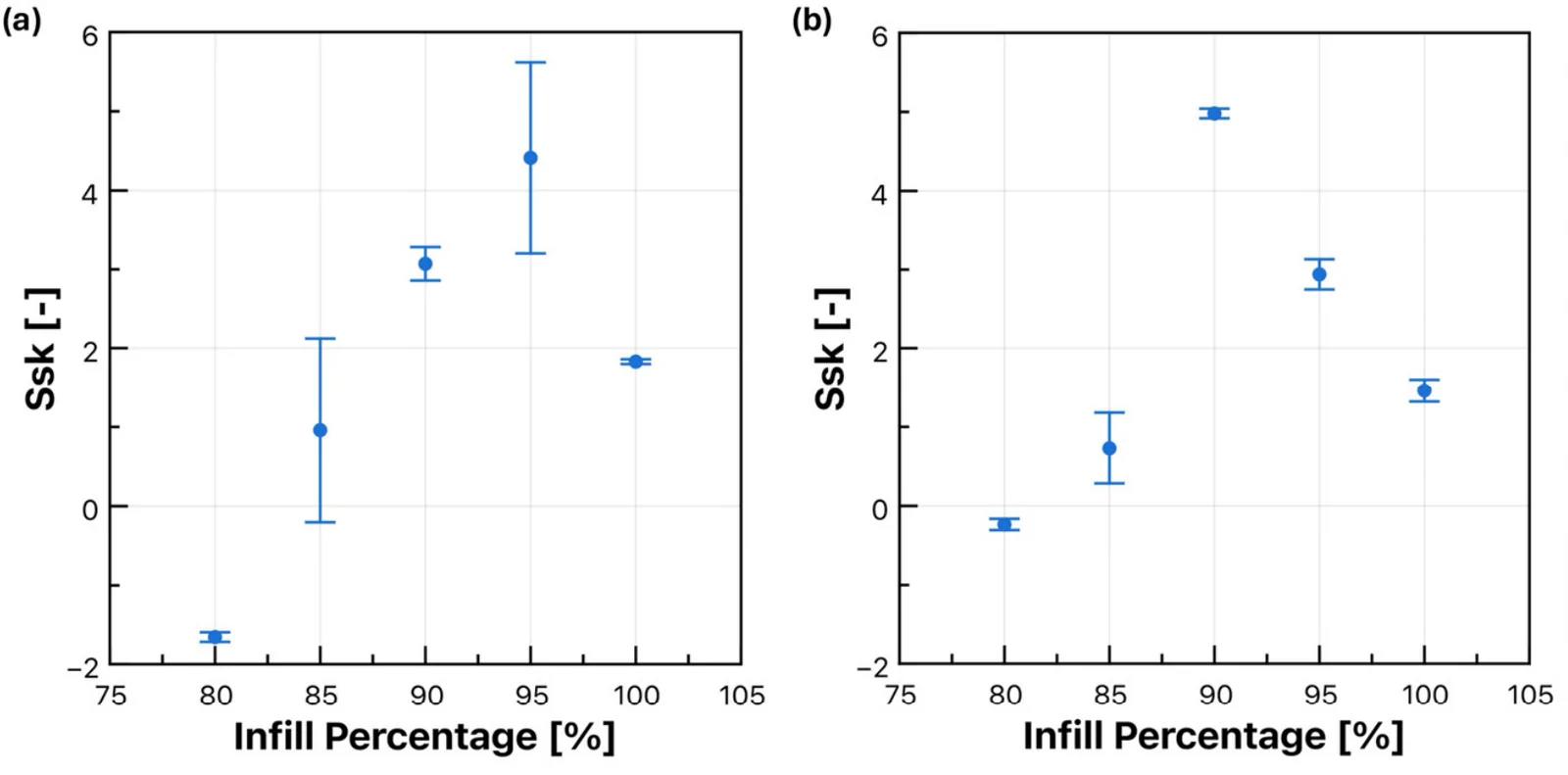
Ssk parameter values as functions of infill percentages for the samples produced with 0.30 mm (**a**) and 0.35 mm layer height (**b**).

**Figure 10:**
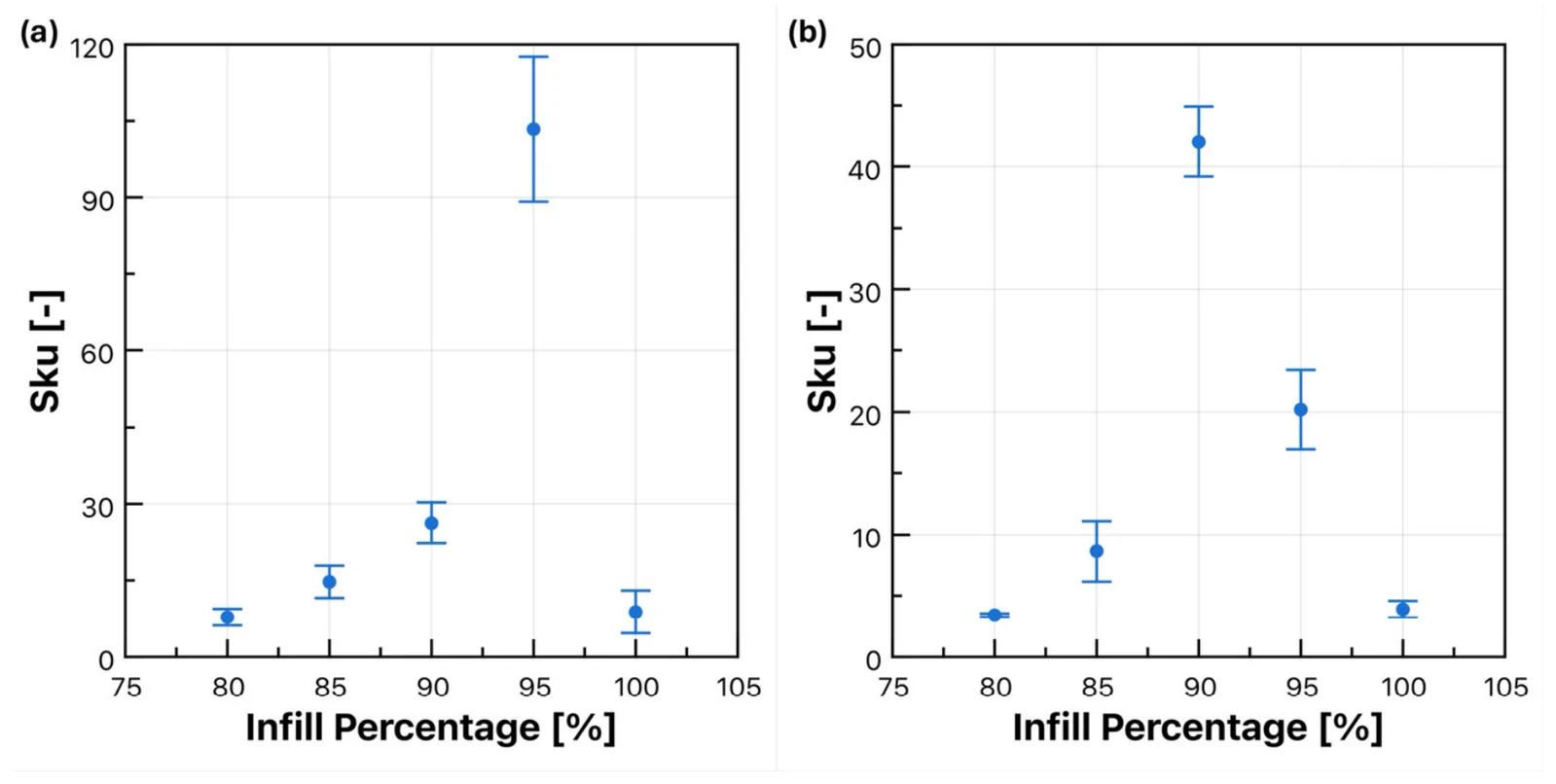
Sku parameter values as functions of infill percentages for the samples produced with 0.30 mm (**a**) and 0.35 mm layer height (**b**).

The Ssk and Sku parameters are an important complement to classical roughness measures, as they describe not so much the amplitude of the irregularities themselves as the statistical shape of the surface elevation distribution. Figure 9 and Figure 10 present the relationships between the infill percentage and Ssk and Sku parameters, respectively. The Ssk parameter (skewness) provides information about the asymmetry of the topography: positive values indicate a predominance of peaks, while negative values indicate a predominance of valleys, which can be significant with regard to the initiation of contact and the distribution of pressures. In turn, Sku (kurtosis) allows to describe the the height distribution and to assess whether the surface is more regular or contains local, distinctly marked extremes. In the analysis of possible tribological surfaces, both parameters are particularly important because they influence the contact mechanism, lubricant retention, and wear development. In the tested samples, the highest values for both Ssk and Sku were obtained at a packing density of 90–95%, whereas for lower packing densities and at 100%, these parameters were lower. This trend suggests that in the intermediate compaction range, the surface exhibits the most pronounced and, at the same time, the most varied statistical character, associated with the presence of local elevations and a sharper topographic profile. For lower degrees of filling, the structure remains more open and less homogeneous, but does not generate such strongly pronounced extremes. At 100% infill percentage excessive path density leads to a more uniform profile and a partial flattening of the height distribution.

**Figure 11:**
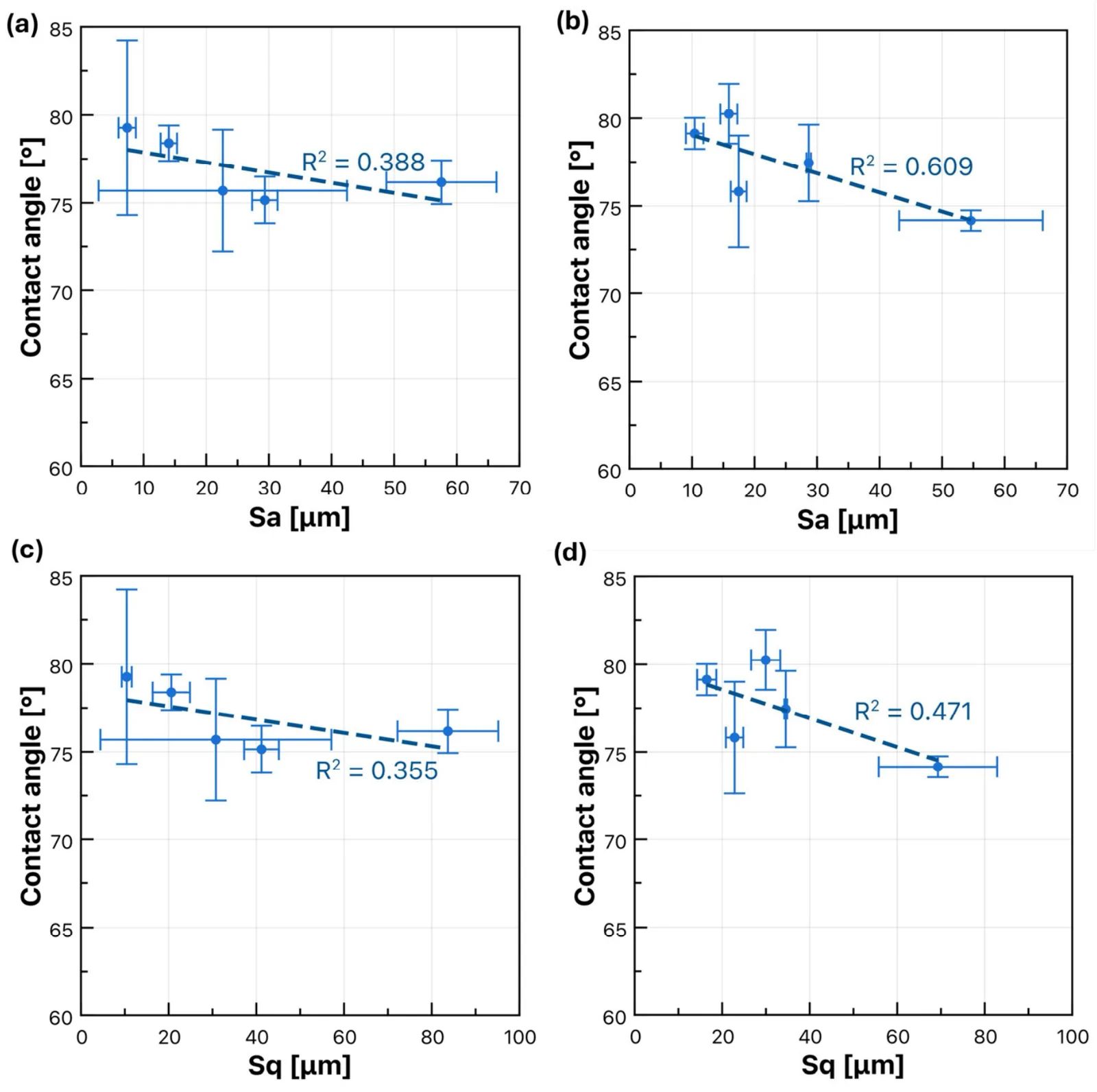
Relationship between the contact angle and roughness parameters—Sa (**a**,**b**) and Sq (**c**,**d**) for the samples produced with the layer height of 0.30 mm (**a**,**c**) and 0.35 mm (**b**,**d**).

The graphs displayed in Figure 11 illustrate the relationship between the contact angle and the fundamental roughness parameters—Sa and Sq. In the analyzed samples, a decreasing relationship was identified between the water contact angle and the roughness parameters Sa and Sq. This means that as the average surface roughness amplitude increased, a systematic decrease in the contact angle was observed, indicating an increase in the effective wettability of the tested surfaces. This trend is consistent with Wenzel’s classical description, according to which surface development enhances the surface’s inherent wetting behavior. For hydrophilic substrates—such as those characterized for the tested PET-G samples—an increase in surface roughness leads to a decrease in the contact angle, as the liquid penetrates the microstructure more easily and increases the effective contact area with the solid material [31]. The literature also emphasizes that, in the case of polymer surfaces, the effect of roughness on the contact angle can be very pronounced but, at the same time, ambiguous, since it depends on the scale of the irregularities, the surface chemistry, and the nature of the contact between the droplet and the substrate. Therefore, this relationship is very often approximate and not fully linear [18]. The linear approximations obtained in this study confirm the existence of a general trend. Coefficients of determination R^2^ ranging from 0.388 to 0.609 indicate that roughness explains a moderate correlation in the variation in the contact angle. Other factors, for example local topographic heterogeneity, the distribution of micro- and macrostructures, and potential differences in the surface’s energy state, may also influence the observed results [18]. In practice, this means that an increase in Sa and Sq promotes improved wettability but does not determine it exclusively, which is consistent with reports on rough surfaces where the Wenzel and Cassie-Baxter mechanisms can coexist, and the experimentally observed behavior depends on whether the droplet penetrates the valleys of the topography or rests partially on trapped air pockets. From the perspective of potential tribological applications, this relationship can be particularly important, as a lower contact angle may promote better retention of the lubricant and more uniform distribution of the lubricating film, while at the same time, greater roughness may increase the proportion of asperity contact and influence friction and wear mechanisms [32]. Therefore, the interpretation of the results requires consideration of the compromise between favorable wetting and a potential increase in mechanical interactions at the interface.

**Figure 12:**
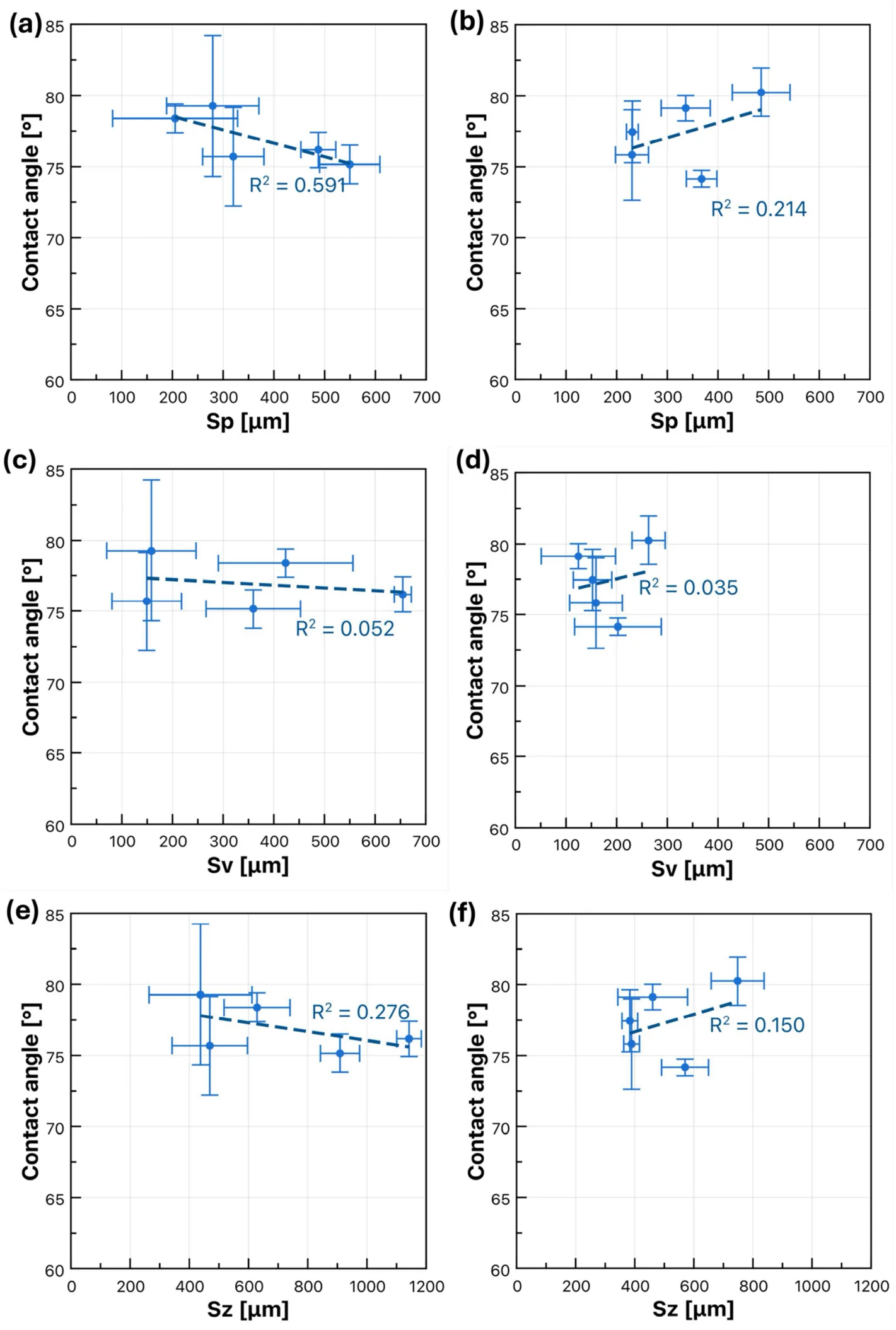
Relationship between the contact angle and roughness parameters—Sp (**a**,**b**) and Sv (**c**,**d**) and Sz (**e**,**f**) for the samples produced with the layer height of 0.30 mm (**a**,**c**,**e**) and 0.35 mm (**b**,**d**,**f**).

Figure 12 depicts the relationships between observed contact angles and Sp, Sv and Sz parameters. The parameters Sp, Sv, and Sz show a very low or moderate degree of correlation with the wettability, and in practice, there is no clear relationship between these quantities, which is confirmed by the low values of the correlation coefficient R^2^ (from 0.035 to 0.591). This means that changes related to the total topographic height, the height of the highest peaks, and the depth of the lowest valleys do not unambiguously correspond to the wetting characteristics of the studied surfaces. This result suggests that the contact angle is more strongly influenced by other structural features—primarily the local organization of surface irregularities, their spatial distribution, and the microscale homogeneity of the surface—rather than by the extreme elevation values themselves. Consequently, the analyzed elevation parameters may be relevant from the perspective of general topography and contact mechanics, but they do not serve as a good indicator for predicting wetting behavior.

**Figure 13:**
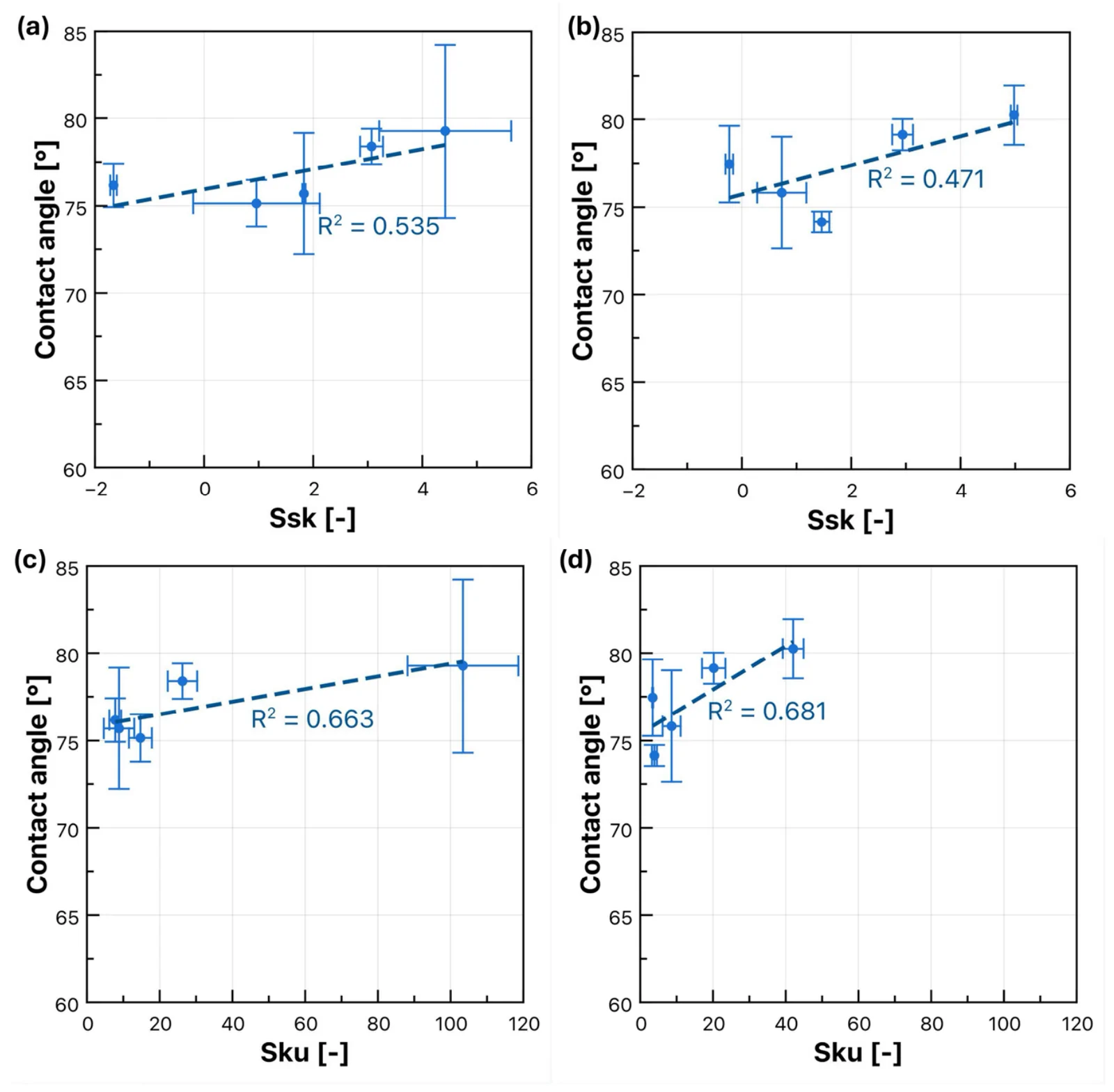
Relationship between the contact angle and roughness parameters—Ssk (**a**,**b**) and Sku (**c**,**d**) for the samples produced with the layer height of 0.30 mm (**a**,**c**) and 0.35 mm (**b**,**d**).

The Ssk and Sku parameters serve as an important complement to classical roughness measures, describing the statistical shape of the surface elevation distribution. The Ssk parameter determines the skewness of the topography and allows one to distinguish between surfaces dominated by peaks and those dominated by valleys, while Sku describes kurtosis—the degree of sharpness of the distribution and the presence of local extremes. In the samples studied, for both layer thicknesses, a linear relationship was observed between the contact angle and the Ssk and Sku parameters; as both parameters increased, the contact angle also increased, and the R^2^ values ranged from 0.471 to 0.681 which indicate moderate correlation of these parameters with the observed contact angles. This means that surfaces with a more positive skewness and higher kurtosis exhibited weaker effective wettability, which can be interpreted as a result of the more pronounced presence of local elevations and a sharper height distribution, limiting drop spreading and favoring less homogeneous contact between the liquid and the substrate. The observed trend is consistent with the classical description of wetting on rough surfaces, according to which topography not only increases the effective contact area but also modifies the way the liquid fills microstructures. As Ssk and Sku increase, the proportion of more asymmetric and more locally concentrated topographic features rises, which may contribute to a partial restriction of liquid penetration into valleys and to the stabilization of droplet contact at the structure’s peaks. Consequently, the observed increase in the contact angle can be attributed to the system transitioning toward a more heterogeneous contact state, in which the liquid does not uniformly fill the entire microgeometry but remains partially supported on more pronounced asperities. From the perspective of possible tribological applications, this result can have important implications, as a higher contact angle may potentially hinder the uniform spreading of the liquid film but, at the same time, promote its localized retention in specific microgeometric regions. This means that the Ssk and Sku parameters can be considered useful auxiliary indicators for assessing the potential functionality of a surface, especially when the goal is to control interactions with a liquid under operating conditions. In practice, this confirms that, in addition to average amplitude parameters, the statistical shape of the height distribution is also important for considering a surface’s wetting behavior. The relationships between the contact angle and Ssk and Sku parameters are presented in Figure 13.

## 4. Conclusions

The analyses conducted demonstrated that the topography and wettability properties of the surfaces of PET-G samples produced by 3D printing are strongly determined by a combination of process parameters, with these relationships often being nonlinear and requiring a multiparameter interpretation. Microscopic observations confirmed the presence of directional material paths, their gradual densification as the fill rate increases, and a general absence of structural defects, which indicates the proper quality of the manufacturing process. For the amplitude parameters Sa and Sq, a nonlinear relationship was observed, in which a decrease in roughness was first observed as the fill factor increased, followed, at full filling, by a renewed increase, interpreted as the effect of excessive track compaction, local material overlap, and accumulations forming during the flow of the plasticized polymer. Measurements of the water contact angle revealed that all surfaces were moderately hydrophilic and quickly reached a steady state, suggesting minimal dynamics in the subsequent spread of the droplet and limited variability in surface properties over time. A statistical analysis involving the relationship between printing parameters such as infill percentage and layer height and the measured contact angle revealed that infill percentage has a significant effect on the observed contact angle, whereas no significant relationship was found for layer height. Within the investigated processing conditions, neither 0.30 nor 0.35 mm can be considered universally preferable; in particular, layer height did not have a statistically significant effect on contact angle according to the two-way ANOVA. Within the investigated parameter set the analysis of the correlation between the contact angle and roughness parameters showed that an increase in Sa and Sq led to a decrease in the contact angle, and thus to an increase in effective wettability; however, moderate values of the coefficient of determination indicate that roughness is not the sole factor determining droplet behavior, but rather interacts with the local geometry of microstructures, the distribution of irregularities, and the potential energy state of the surface. In contrast, the parameters Sp, Sv, and Sz showed no clear correlation with the wetting angle, which means that extreme topographic values have limited predictive value for describing wettability, and that parameters describing the overall shape and organization of the elevation distribution are more important factors. In this context, the following parameters proved to be particularly important: Ssk and Sku, for which a linear relationship with the wetting angle was observed: as both parameters increased, so did the wetting angle, indicating a deterioration in wettability for surfaces with a more positive slope and greater kurtosis—that is, surfaces that are more asymmetric and sharp with a predominance of distinct local elevations. The observed associations between Ssk, Sku, and contact angle suggest that these surface topography parameters may be relevant to the wettability behavior of 3D-printed PET-G; however, their predictive capability cannot be established based on the present dataset. Ssk and Sku provided additional descriptors of the surface morphology and showed associations with wettability; however, their applicability as process-monitoring parameters was not established in the present study. The changes in water wettability observed for the investigated PET-G surfaces may be relevant to their potential use in tribological applications; however, these implications remain hypothetical and require direct evaluation using relevant lubricants and tribological testing.

## 5. Limitations and Further Research

In future studies, it would be valuable to extend the analysis to include detailed surface chemical characterization and surface-energy measurements using a broader range of appropriately selected test liquids. In particular, further investigations using liquids with different physicochemical properties, including non-polar liquids, as well as dynamic contact-angle measurements, could provide a more comprehensive understanding of the respective contributions of surface chemistry and topography to the observed wettability behavior. External validation using independent specimens produced under additional and previously untested processing conditions will be required to assess the robustness and general applicability of the relationships identified in the present study. It would also be valuable to consider using a broader set of spatial topography parameters, multiscale analyses, and numerical models to better describe the relationship between microgeometry and the wetting mechanism. This should be further supplemented by tribological studies, which would enable a direct link between the observed surface features and actual behavior under friction and wear conditions, thereby allowing for a more comprehensive assessment of the functionality of the analyzed structures in engineering applications. Future studies should include a broader range of printing conditions and a larger experimental dataset to further investigate the relationships between surface topography parameters and wettability and to determine whether Ssk and Sku can provide robust predictive information.

## Figures and Tables

**Table 1:** Samples designations with layer heights and infill percentages values.

Sample	Layer Height [mm]	Infill [%]
0.30/80	0.30	80
0.30/85	0.30	85
0.30/90	0.30	90
0.30/95	0.30	95
0.30/100	0.30	100
0.35/80	0.35	80
0.35/85	0.35	85
0.35/90	0.35	90
0.35/95	0.35	95
0.35/100	0.35	100

**Table 2:** Contact angle values for the produced samples.

Sample	Contact Angle [°]
0.30/80	76.17 ± 1.24
0.30/85	75.15 ± 1.34
0.30/90	78.39 ± 1.01
0.30/95	79.27 ± 4.96
0.30/100	75.69 ± 3.47
0.35/80	77.45 ± 2.18
0.35/85	75.82 ± 3.19
0.35/90	80.25 ± 1.70
0.35/95	79.13 ± 0.89
0.35/100	74.15 ± 0.60

**Table 3:** The ANOVA table from the two-way ANOVA considering two factors with the interaction term.

Condition	Sum of Squares	df	Mean Square	F	*p*-Value
Infill [%]	101.438	4	25.359	4.310	0.0112
Layer height [mm]	1.532	1	1.532	0.260	0.6154
Infill × Layer height	10.248	4	2.562	0.435	0.7815
Error	117.675	20	5.884		
Corrected total	230.893	29			

**Table 4:** The post-hoc Tukey’s tests results.

Comparison	Mean Difference	*p* Tukey	Significance
85% vs. 90%	3.83°	0.0499	Yes
90% vs. 100%	4.40°	0.0203	Yes
95% vs. 100%	4.28°	0.0239	Yes
Others	-	>0.05	No

## Data Availability

The original contributions presented in this study are included in the article. Further inquiries can be directed to the corresponding author.

## Abbreviations

The following abbreviations are used in this manuscript:

FDM	Fused Deposition Modeling
FFF	Fused Filament Fabrication
PET-G	Polyethylene Terephthalate Glycol
